# Supplementary material for: MLN4924 Treatment Diminishes Excessive Lipid Storage in High-Fat Diet-Induced Non-Alcoholic Fatty Liver Disease (NAFLD) by Stimulating Hepatic Mitochondrial Fatty Acid Oxidation and Lipid Metabolites
Source: Pharmaceutics. 2022 Nov 15;14(11):2460. doi: 10.3390/pharmaceutics14112460 (PMC9696831; doi:10.3390/pharmaceutics14112460)
Supplement: Supplementary file 1 [file pharmaceutics-14-02460-s001.zip › pharmaceutics-2001220-supplementary.pdf]

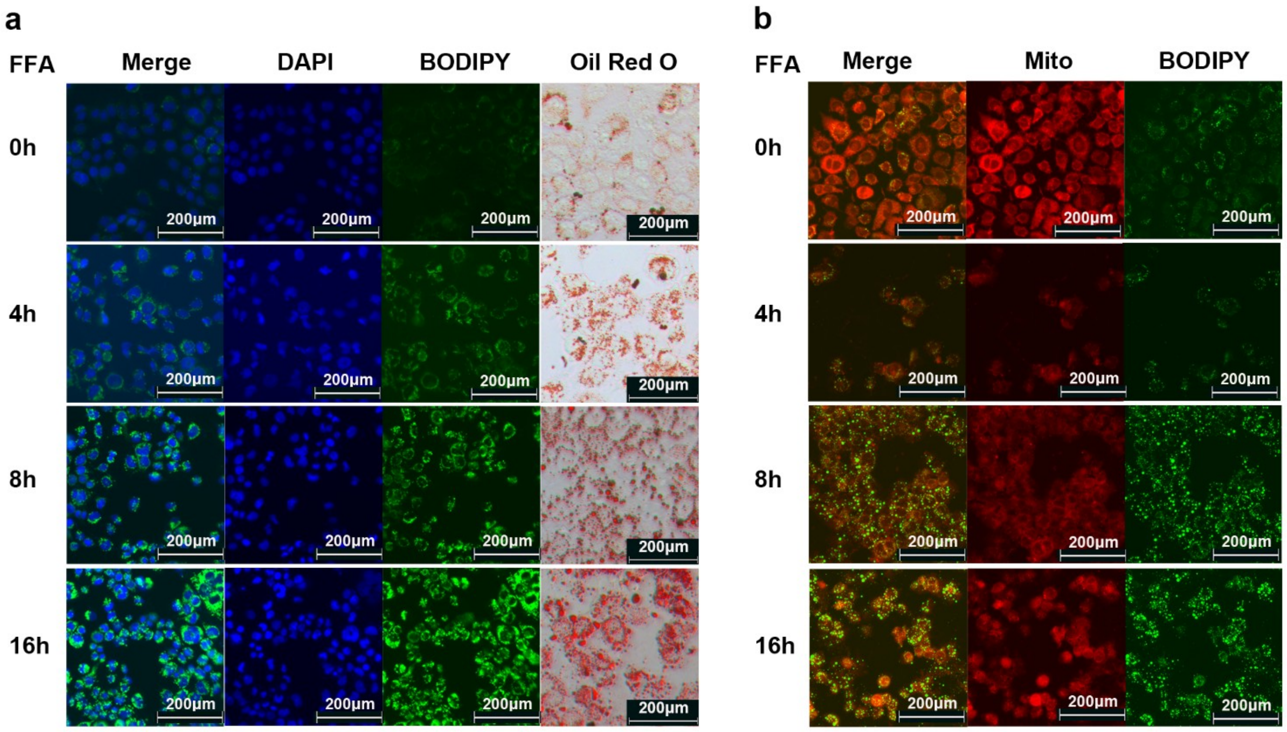

Figure S1. Lipid accumulation can be induced by free fatty acid in Huh7 cells. Huh7 cells were treated with 1mM FFA for 0, 4, 8, 16 hours. **(a)** Representative images of DAPI, BODIPY and oil red O (ORO). **(b)** Representative images of MITORED and BODIPY. Scale bar: 200μm.

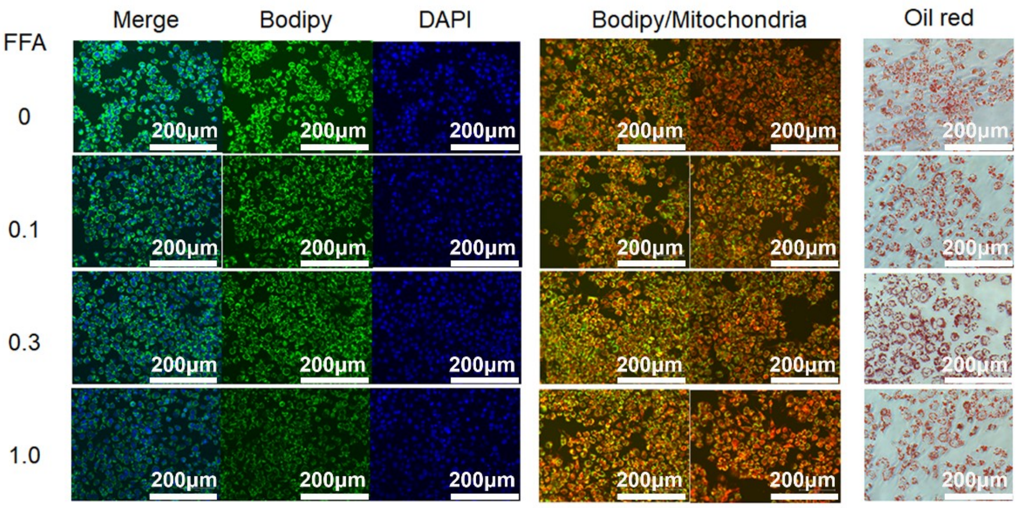

Figure S2. Neddylation inhibitor MLN4924 can inhibit lipid accumulation induced by free fatty acid in Huh7 cells. Huh7 cells were treated with 0, 0.1, 0.3 or 1μM MLN4924 and 1mM FFA for 16 hours. Representative images of DAPI, BODIPY and oil red O (ORO). Representative images of MITORED and BODIPY. Scale bar:200μm.

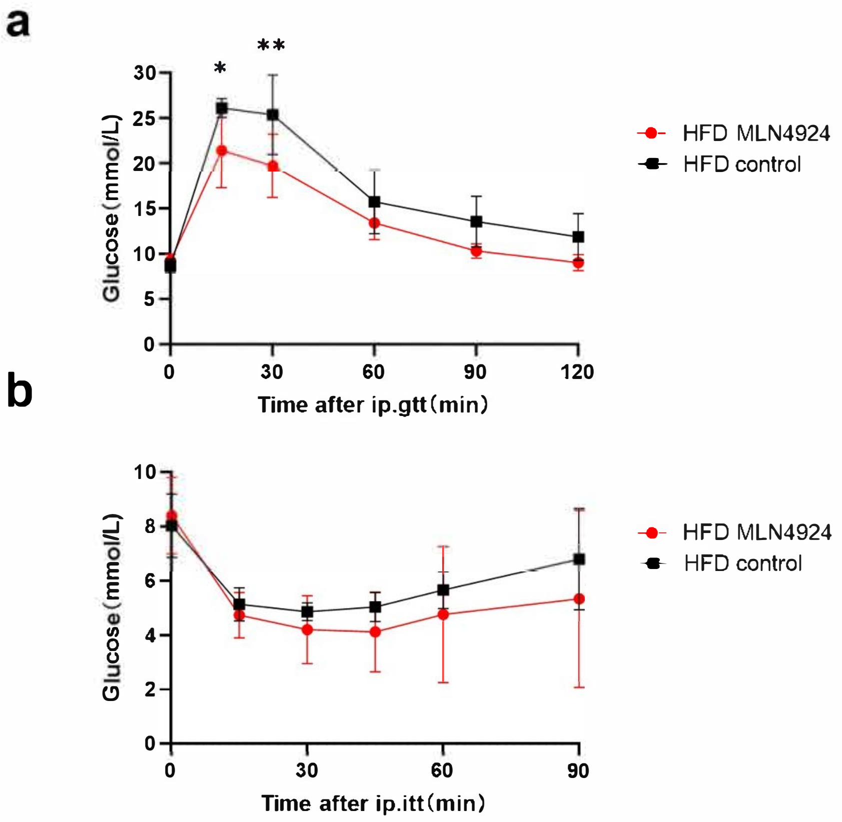

Figure S3. MLN4924 ameliorates insulin resistence in mice. **(a,b)** GTT (glucose tolerance test) and ITT (insulin tolerance test) were measured after 12-week HFD (n = 5 per group). Two-way ANOVA was used for statistical analysis (*$P < 0.05$, **$P < 0.01$).

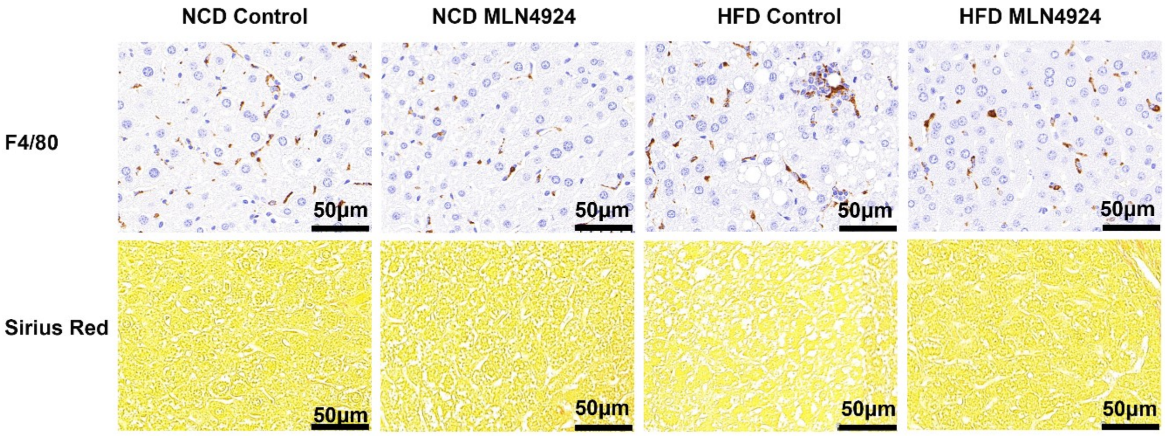

Figure S4. MLN4924 affects the inflammation of liver in mice. Representative immunohistochemical staining pictures of liver sections from NCD-fed and HFD-fed male mice treated with or without MLN4924 showed the levels of F4/80. Representative images of Sirius Red staining liver tissues in indicated mice were presented.

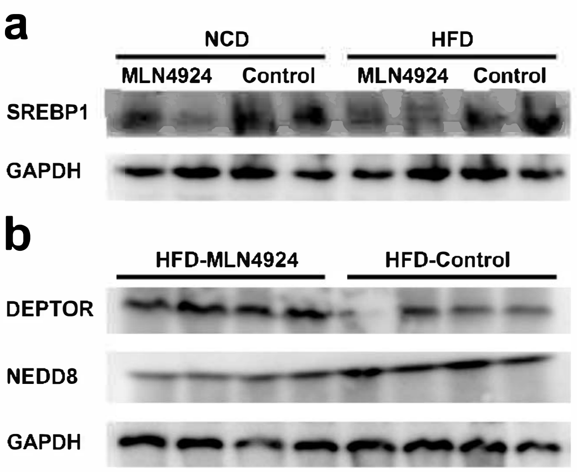

Figure S5. MLN4924 change the expressions of SREBP1 and DEPTPOR in mice. **(a,b)** Representative images of western blot showed the effects of MLN4924 on SREBP1, DEPTPOR and NEDD8 expression. GAPDH was used as an invariant control.
